# Weighting climate model projections using observational constraints

**DOI:** 10.1098/rsta.2014.0425

**Published:** 2015-11-13

**Authors:** Nathan P. Gillett

**Affiliations:** Canadian Centre for Climate Modelling and Analysis, Environment Canada, University of Victoria, PO Box 1700, STN CSC, Victoria, British Columbia, Canada V8W 2Y2

**Keywords:** climate projections, Transient Climate Response, observational constraints

## Abstract

Projected climate change integrates the net response to multiple climate feedbacks. Whereas existing long-term climate change projections are typically based on unweighted individual climate model simulations, as observed climate change intensifies it is increasingly becoming possible to constrain the net response to feedbacks and hence projected warming directly from observed climate change. One approach scales simulated future warming based on a fit to observations over the historical period, but this approach is only accurate for near-term projections and for scenarios of continuously increasing radiative forcing. For this reason, the recent Fifth Assessment Report of the Intergovernmental Panel on Climate Change (IPCC AR5) included such observationally constrained projections in its assessment of warming to 2035, but used raw model projections of longer term warming to 2100. Here a simple approach to weighting model projections based on an observational constraint is proposed which does not assume a linear relationship between past and future changes. This approach is used to weight model projections of warming in 2081–2100 relative to 1986–2005 under the Representative Concentration Pathway 4.5 forcing scenario, based on an observationally constrained estimate of the Transient Climate Response derived from a detection and attribution analysis. The resulting observationally constrained 5–95% warming range of 0.8–2.5 K is somewhat lower than the unweighted range of 1.1–2.6 K reported in the IPCC AR5.

## 1. Background

Projections of climate change, as presented for example in the Fifth Assessment Report of the Intergovernmental Panel on Climate Change (IPCC AR5), are typically derived from the available set of climate models, with uncertainties based on inter-model spread [1]. However, this ensemble may be biased; for example, some models from the Fifth Coupled Model Intercomparison Project (CMIP5) appear to have a response to greenhouse gas increases which is too strong compared with observations [2]. Moreover, model spread may not be an appropriate measure of uncertainty in climate projections: Annan & Hargreaves [3] demonstrate that model spread is only a valid measure of uncertainty under the ‘indistinguishable’ case where the real-world and climate models are drawn from the same distribution. Though this assumption may be reasonable based on current knowledge, as the signal of anthropogenic climate change strengthens it is to be expected that climate system properties such as the Transient Climate Response (TCR; defined as the warming averaged over a 20-year period at doubled pre-industrial CO_2_ concentration in a simulation with CO_2_ increasing at 1% per annum) may be increasingly well constrained from observations [4]. If observed transient climate change continues not to be used to tune climate models [5], then observationally constrained estimates of parameters such as TCR are likely to become progressively narrower than the model range. On the other hand, if transient climate change were used to tune climate models, it would be expected that models’ climate system properties such as TCR would converge on observational estimates by construction.

For the first time, the most recent IPCC assessment included an estimate of near-term warming based not only on the raw CMIP5 simulations but also on an observationally constrained estimate. This was derived by scaling projected warming in response to greenhouse gas increases and aerosol changes by the regression coefficients of observed temperature change onto the simulated responses to greenhouse changes and aerosol changes, following the Allen *et al.* [6] and Stott & Kettleborough [4] (ASK) approach [7,8]. The observationally constrained estimate of near-term warming was somewhat lower and more closely constrained than the raw CMIP5 model output, suggesting that the most strongly warming CMIP5 projections in the near term could be ruled out [8]. However, while the ASK approach works well for near-term projections under conditions of progressively increasing radiative forcing, it works less well for long-term projections and under conditions of stabilized radiative forcing. A comparison of energy balance model simulations with varying climate sensitivity and ocean diffusivity found that while the assumption of a linear relationship between historical warming and future warming is reasonably good for scenarios with progressively increasing forcing, this assumption is less good for a stabilization scenario [9]. This is because two climate models with different climate sensitivities and ocean diffusivities, or equivalently a climate model and the real world, may have similar responses during a period of progressively increasing forcing, but diverging responses after stabilization of radiative forcing. Hence this approach was not applied to long-term projections in the IPCC AR5. Nonetheless, we might still expect for example that climate models having a TCR significantly higher than that constrained from observations may tend to overestimate warming over the longer term. A number of studies have proposed approaches to deriving probabilistic global and regional climate projections by applying observational constraints to perturbed physics ensembles [10–12]. Such studies have the advantage that they more systematically sample over uncertainties in model parameter space, but results may be hard to compare with projections derived from raw climate model projections as reported, for example, in the IPCC AR5. Here we propose a simple approach to weighting climate model projections based on their agreement with an observed metric, in order to derive simple observationally constrained probabilistic projections.

## 2. Method and results

The first step in deriving observationally constrained projections is the identification of an appropriate observable metric to be used as a model constraint. The metric identified needs to be reliably estimated from observations, with well-constrained uncertainties, and it needs to be well correlated with the predictand across the model ensemble. Note that a perfect correlation between the metric and predictand is not required: this would imply a linear transfer function, and might be more straightforwardly approached using a scaling approach such as ASK. In this example, we will focus on projections of 2081–2100 temperature constrained based on an observational estimate of TCR [13]. Although the TCR is not a directly observable parameter, it is one for which multiple observationally constrained estimates exist [2], and one which might be expected to correlate well with projected warming. A similar approach could in principle be applied to any observable quantity which is correlated with a predictand of interest, across a model ensemble.

**Figure 1.**
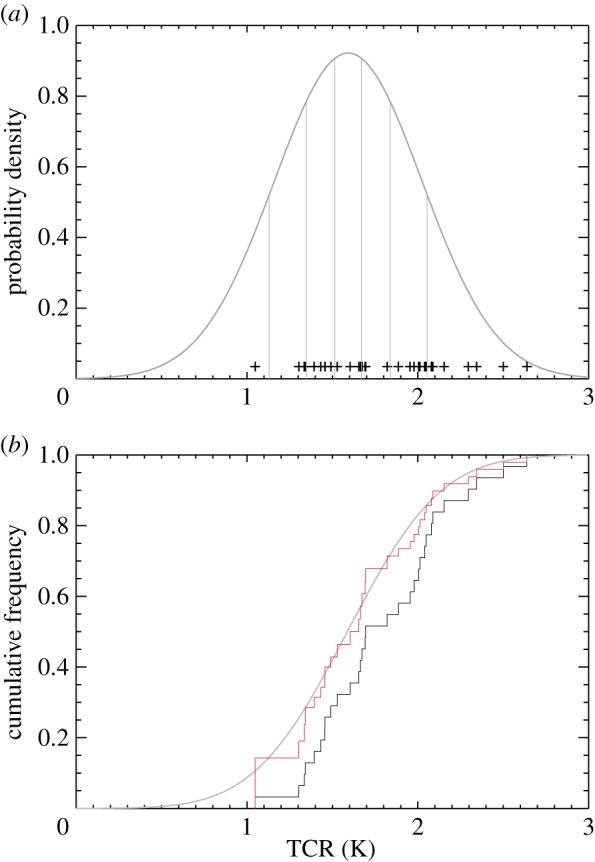
A comparison of an observationally constrained distribution of TCR with a distribution derived from the CMIP5 models. (*a*) A PDF of TCR, estimated by fitting a normal distribution to the mean and 5–95% range estimated from observations [13] (grey curve), is compared with the TCR of 31 CMIP5 models (black crosses). Vertical grey lines divide the PDF into seven equal-area quantiles. (*b*) An observationally constrained cumulative distribution function of TCR (grey curve), corresponding to the PDF in (*a*), is compared with cumulative frequency distributions of TCR derived from unweighted CMIP5 model TCRs (black curve), and derived by giving equal weight to the model TCRs lying in each of the seven quantiles shown in (*a*) (red curve).

Crosses in figure 1*a* show the TCR of 31 CMIP5 models for which the required data are available. These are compared with an observationally constrained estimate of TCR from Gillett *et al.* [13] which was derived from a detection and attribution analysis (The probability density function (PDF) shown is a normal distribution fitted to the mean and 5–95% range reported by Gillett *et al.* [13]. Such a distribution is approximately consistent with their assumptions.) Other observationally constrained estimates of TCR are available, which differ based on the data and approach used, though most have a mean lower than the mean TCR of the CMIP5 models [1,2]. Results are expected to be somewhat sensitive to the particular observational estimate of TCR used. As shown in figure 1*a*, the range of TCRs of the CMIP5 models is not centred on the mean of the observational estimate but lies towards the higher end of the distribution [1]. Figure 1*b* compares the corresponding cumulative distribution function of TCR based on Gillett *et al.* [13] (grey curve) with a cumulative frequency distribution derived from CMIP5 models (black curve) as follows. If *T*_*i*_ are the model TCRs sorted in ascending order then a simple cumulative frequency distribution may be defined
2.1
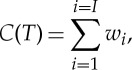
where *I* is chosen such that *T*_*I*_<*T*<*T*_*I*+1_ and *w*_*i*_=1/*N* where *N* is the number of models. This simple cumulative distribution function weights each model equally. Note that a more realistic estimate of the cumulative density function would be greater than 0 for *T*<*T*_1_ and less than 1 for *T*>*T*_*N*_ but we use this simple function since its standard deviation is well defined.

We may then ask whether we can weight models differently in order to bring the model-based distribution of TCR into closer agreement with the observational estimate. A simple approach to doing this is to find the maximum number of quantiles, *M*, into which the observed PDF can be divided with at least one model TCR in each quantile. For the example shown in figure 1*a*, *M*=7 and quantile boundaries are shown by the vertical grey lines. If we now define
2.2
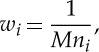
where *n*_*i*_ is the total number of model TCRs lying in the same quantile as *T*_*i*_, and substitute into equation (2.1), we obtain the red cumulative frequency distribution shown in figure 1*b*. This approach gives equal weight to models lying in each quantile of the observational PDF. In the example shown in figure 1*a* there is a single model lying in the lowest of seven quantiles, so its TCR is given weight 1/7, while there are seven models lying in the highest quantile, so each of their TCRs is given weight 1/(7×7). By construction the derived distribution agrees exactly with the observational distribution at the quantile boundaries (shown by vertical grey lines in figure 1*a*). Other measures of the distribution, such as the mean, variance and particularly extremes, are not expected to agree exactly with the observational distribution. However, as the number of quantiles, *M*, increases agreement is expected to improve.

**Figure 2.**
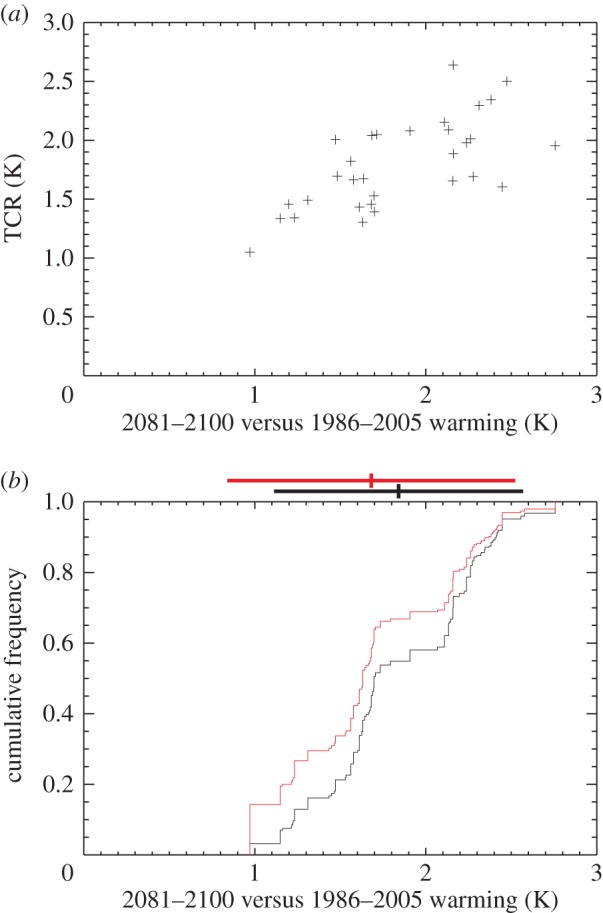
Application of model weighting to projected warming. (*a*) A scatter plot of CMIP5 model TCR against ensemble mean warming in 2081–2100 relative to 1986–2005 for the 31 CMIP5 models considered in this study (*r*=0.68). (*b*) Cumulative frequency distributions of warming in 2081–2100 relative to 1986–2005 derived by giving equal weight to each of 31 CMIP5 models (black curve), and derived by giving equal weight to the models whose TCRs lie in each of the seven quantiles of observed TCR shown in figure 1*a* (red curve). Black and red confidence bars above the plot show means and 5–95% confidence ranges calculated from the means and standard deviations of the raw model and observationally constrained distributions, respectively [1].

While deriving an improved model-based distribution of TCR is not in itself useful, given that we already have an observationally constrained estimate of this quantity, the model weights derived using this constraint can be applied to climate change projections for which we do not have observational estimates. Figure 2*a* shows a scatterplot of CMIP5 model TCR for the same 31 models against ensemble mean projected 2081–2100 warming relative to a 1986–2005 base period under the Representative Concentration Pathway (RCP) 4.5 scenario [1]. As expected these quantities are correlated (*r*=0.68), but the correlation is not perfect. Besides a contribution from internal variability, two other factors contribute to the spread. First, while TCR represents only the response to CO_2_ change for linearly increasing forcing, projected warming in RCP 4.5 is a response to a range of forcings including changes in multiple greenhouse gases and aerosols, and the relative responses to each forcing may differ between models. Second, as already discussed, the assumption of a linear relationship between historical warming and future warming is less good for a stabilization scenario such as RCP 4.5 [9]. For these reasons, observationally constrained projections were not used in assessments of projected warming for the period past 2035 in the IPCC AR5 [1].

We start by deriving a cumulative frequency distribution in which each model has equal weight, using output from all available simulations, since models have ensembles of simulations available with up to ten members. If the Δ*T*_*j*_ are the warmings simulated in individual ensemble members from all models sorted in ascending order, then the cumulative frequency distribution may be defined as
2.3
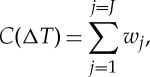
where
2.4
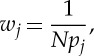
*J* is chosen such that Δ*T*_*J*_<Δ*T*<Δ*T*_*J*+1_, and *p*_*j*_ is the size of the ensemble from which the *j*th simulation is drawn. Again note that this cumulative frequency distribution is 0 for Δ*T*<Δ*T*_0_ and 1 for Δ*T*>Δ*T*_*N*_, which is desirable as we wish to calculate its standard deviation. This distribution is shown in black in figure 2*b*. This approach uses all available ensemble members in deriving the cumulative frequency distribution, and gives equal weight to each model. Collins *et al.* [1] report a 5–95% range of 1.1–2.6 K for 2081–2100 warming in RCP 4.5 which they derived by taking a single ensemble member from each of 42 CMIP5 models, calculating the mean and standard deviation across the multi-model ensemble and multiplying the standard deviation by 1.64 based on the assumption of a Gaussian distribution. Using updated CMIP5 data from a subset of 31 models for which TCR could also be calculated, and using all available ensemble members, with equal weight assigned to each model when deriving the mean and standard deviation as in equation (2.4), we also obtained a 5–95% range of 1.1–2.6 K for RCP 4.5 warming in 2081–2100 (shown by the black bar at the top of figure 2*b*).

If we now weight models using our observational constraint on TCR, defining
2.5
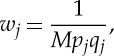
where *q*_*j*_ is the number of models whose TCR falls in the same quantile of the observational TCR distribution as that from which the *j*th simulation is obtained, and substituting in equation (2.4), we obtain the red cumulative frequency distribution shown in figure 2*b*. As expected, given that the mean observational TCR is lower than the mean model TCR, and the positive correlation between TCR and projected warming, this distribution has a lower mean (1.7 K) than the unweighted model distribution (1.8 K). The red confidence bar at the top of figure 2*b* shows the 5–95% range of 0.8–2.5 K estimated from the mean and standard deviation of the observationally constrained distribution following the approach of Collins *et al.* [1], which as expected is also lower than the corresponding unweighted range. Such a reduction in projected warming when accounting for observational constraints is broadly consistent with near-term climate projections as assessed in the IPCC AR5: whereas the CMIP5 model 5–95% range for 2016–2035 warming relative to 1986–2005 is 0.47–1.00 K, an observationally constrained estimate of warming for this period, derived using the ASK approach [8], is 0.39–0.87 K [7]. For comparison, the 5–95% range derived using the TCR-weighting approach described here applied to RCP 4.5 is 0.37–0.99 K.

## 3. Discussion and conclusion

The approach described relies first on having a large number of models whose observable quantities (in this case TCR) fully span the observed distribution of this quantity with sufficient density such that the observed PDF can be divided into as large a number of quantiles as possible. In the example shown in figure 1*a*, the lowest model TCR lies in the 10th percentile of the observed distribution, suggesting that the low part of the range of TCR may be under-sampled by the CMIP5 models. This may be a particular problem for estimates of the low extremes of the weighted distribution of projections: clearly, the lowest warming in the weighted cumulative frequency distribution will never be lower than the lowest simulated warming in the CMIP5 models themselves, and similarly for the highest warming. On the other hand, there may be physical constraints on the lower bound of TCR and projected warming reflected in the CMIP5 models, but not in the observationally constrained estimate of TCR, in which case these will be reflected in projections derived using this approach. This requirement does, however, imply that in order for multi-model projections to span the full range of uncertainties consistent with observations, climate models should not be tuned to reproduce an observational best estimate of TCR or other metrics of transient climate change. Second, the approach described requires an accurate PDF of the observable, which should be as narrow as possible and as highly correlated with the predictand as possible.

The example described here used an observational estimate of TCR as the observational constraint, but the approach could use any observable with well-characterized uncertainties and a high correlation with the predictand. Given that the TCR estimate from Gillett *et al.* [13] was derived from a detection and attribution analysis applied to historical temperature changes, a more direct quantity to use would be the historical greenhouse-gas-attributable warming trend, which has more closely constrained uncertainties than TCR. The practical reason for not using this constraint is that relatively few CMIP5 models carried out the necessary greenhouse gas only historical simulations with which to compare this. It might be thought that simply the rate of warming over the historical period could be used as an observational constraint, but whereas differences in historical warming rates between CMIP5 models are driven mainly by differences in radiative forcing trends (likely dominated by differences in aerosol forcing), future warming rates depend mainly on climate sensitivity differences [14], hence the rates of historical warming and future warming are unlikely to be strongly correlated across the CMIP5 ensemble. The approach could also be applied using a climatological observational constraint which correlates with a projected change, such as using the current seasonal cycle to constrain snow albedo feedback on climate change [15], though further work is required to identify such robust relationships for global climate change variables.

Overall, the approach described here offers a simple way to move beyond reporting means and uncertainty ranges for long-term climate projections based simply on the spread of available unweighted climate model simulations, as was done in the IPCC AR5 [1], by weighting model projections based on an observational constraint. This approach is less sophisticated than some other approaches to deriving observationally constrained climate projections, which sample over multiple sources of uncertainty and consider multiple observational constraints, often using perturbed physics ensembles [10,11]. However, it has the advantage that, since it is simply a re-weighting of existing climate model projections, it is easy to relate to the widely used climate projections presented in the IPCC AR5. As expected when the approach is applied to twenty-first century warming under the RCP 4.5 scenario using a previously published observationally constrained estimate of TCR [13], it gives rise to a somewhat lower mean and range of warming than the raw CMIP5 model projections presented in the IPCC AR5. As observed climate change further intensifies and the number of available climate models increases, this approach may offer a way to derive increasingly closely constrained climate projections in the future.

## Data accessibility

The analysis presented in this paper was based wholly on publicly available CMIP5 data.
